# Supplementary material for: Osteocyte mitochondria regulate angiogenesis of transcortical vessels
Source: Nat Commun. 2024 Mar 21;15:2529. doi: 10.1038/s41467-024-46095-0 (PMC10957947; doi:10.1038/s41467-024-46095-0)

Inventory of Supporting Information

Osteocyte mitochondria regulate angiogenesis of transcortical vessels

Peng Liao[1,2]†, Long Chen[3]†, Hao Zhou[4]†, Jiong Mei[1], Ziming Chen[5,6], Bingqi Wang[1,2], Jian Q. Feng[7], Guangyi Li[1], Sihan Tong[1,2], Jian Zhou[1,2], Siyuan Zhu[8], Yu Qian[9], Yao Zong[5,6], Weiguo Zou[1,2,3], Hao Li[1,2], Wenkan Zhang[4], Meng Yao[1,2], Yiyang Ma[1,2], Peng Ding[1,2], Yidan Pang[1,2], Chuan Gao[1,2], Jialun Mei[1,2], Senyao Zhang[1,2], Changqing Zhang[1,2*], Delin Liu[1,2*], Minghao Zheng[5,6*], Junjie Gao[1,2*]

1. Department of Orthopaedics, Shanghai Sixth People's Hospital Affiliated to Shanghai Jiao Tong University School of Medicine, Shanghai, 200233, China
2. Institute of Microsurgery on Extremities, Shanghai Sixth People's Hospital Affiliated to Shanghai Jiao Tong University School of Medicine, Shanghai, 200233, China
3. State Key Laboratory of Cell Biology, Shanghai Institute of Biochemistry and Cell Biology, CAS Center for Excellence in Molecular Cell Science, Chinese Academy of Sciences, University of Chinese Academy of Sciences, Shanghai, 200031, China
4. Department of Orthopedics, The Second Affiliated Hospital of Zhejiang University School of Medicine, Hangzhou, Zhejiang Province, 310009, China
5. Centre for Orthopaedic Research, Medical School, The University of Western Australia, Nedlands, Western Australia, 6009, Australia
6. Perron Institute for Neurological and Translational Science, Nedlands, Western Australia, 6009, Australia
7. Shanxi Medical University School and Hospital of Stomatology, Shanxi Province Key Laboratory of Oral Diseases Prevention and New Materials, Taiyuan, Shanxi Province, 030001, China
8. Department of General Surgery, Shanghai Sixth People's Hospital Affiliated to Shanghai Jiao Tong University School of Medicine, Shanghai, 200233, China
9. Department of Orthopedics, The First Affiliated Hospital, Zhejiang Chinese Medical University, Hangzhou, Zhejiang Province, 310006, China

Contents	Page
Supplementary Table 1	2
Supplementary Table 2	3-4
Supplementary Figure 1	5
Supplementary Figure 2	6
Supplementary Figure 3	7
Supplementary Figure 4	8
Supplementary Figure 5	9
Supplementary Figure 6	10
Supplementary Figure 7	11
Supplementary Figure 8	12
Supplementary Figure 9	13
Supplementary Figure 10	14
Supplementary Figure 11 Uncropped Western blots.	15

Supplementary Table 1

a. Overlapped genes between downregulated genes related to blood vessel in *Dmp1*^{cre}DTA mice and endothelial cells derived angiogenesis genes in dataset (*p*<0.05)

**Downregulated genes related to vessel in *Dmp1*$^{Cre-}$DTA**					**Overlapped Genes**	**Endothelial cells derived angiogenesis genes in dataset**				
Col1a1	Vegfc	Ramp3	Angpt4	Srpx2		Adamts1	Fgf2	Ccl2	Cxcl12	Notch
Mmp9	Notch4	Col18a1	Adam12	Col4a2	Slit3	Areg	Fgf7	Ccl3	Serpine1	TGFb
Mmp14	Nrarp	Rasip1	Col22a1	Ecm1		Ang	Cx3cl1	Mmp3	Serpinf1	
Fn1	Mfge8	Bcar1	Amotl1	Aldh1a2		Angpt1	Csf2	Mmp8	Thbs2	
Col8a1	Robo4	Vash1	Tie1	Fgfr1		Angptl3	Hbegf	Mmp9	Timp1	
Col5a1	Flt4	Anpep	Stab1	Sox18	VEGFc	F3	Hgf	Ccn3	Timp4	
Col4a1	Pkd1	Angptl4	Pdgfb	Dlx3		Cxcl16	Igfbp1	Spp1	Vegf	
Nrp2	Dll4	Vav2	Adcy6	Unc5b		Ccn1	Igfbp2	Tymp	Vegfa	
Aplnr	Foxp1	Tnfsf12	Foxc1	Eng		Dll4	Igfbp3	Pdgfa	Vegfb	
Plxnd1	Foxc2	Hyal1	Ephb4	Pdgfrb	Notch3	Dpp4	Il1a	Pdgfb	Slit3	
Cldn5	Cspg4	Ihh	Adgra2	Fmnl3		Egf	Il1b	Ptx3	VAO	
Ephb1	Nos3	Ppp1r16b	Vangl2	Hhip		Eng	Il10	Pf4	RANKL	
Slit3	Hoxa5	Heg1	Arhgef26	Ephb3		Col18a1	Cxcl10	Pgf	Noggin	
Notch3	Rapgef3	C6	Hey2	Hoxa3	Notch4	Edn1	Cxcl1	Prl	HIF1alpha	
	Hoxb3	Wnt5a	Ccbe1	Ephb2		Fgf1	Lep	Prl2c2	DLL4	

b. The RT-PCR primers used in this study

**Gene**	**Forward primers**	**Reverse primers**
**mus-GAPDH**	**5’-CAGGTTGTCTCCTGCGACTT-3’**	**5’-TATGGGGGTCTGGGATGGAA-3’**
**mus-VEGFc**	**5’-GAGGTCAAGGCTTTTGAAGGC-3’**	**5’-CTGTCCTGGTATTGAGGGTGG-3’**
**mus-Slit3**	**5’-CTCTGGCCCTCCCTTACAAG-3’**	**5’-GCTACTGAGATAGTGGCCTGAAG-3’**
**mus-Notch3**	**5’-TGCCAGAGTTCAGTGGTGG-3’**	**5’-CACAGGCAAATCGGCCATC-3’**
**mus-Notch4**	**5’-CTCTTGCCACTCAATTTCCCT-3’**	**5’- TTGCAGAGTTGGGTATCCCTG-3’**

Supplementary table 1. (**a**) Overlapping angiogenesis genes between the cortical bone bulk RNA-seq database of *Dmp1*$^{Cre-}$*DTA*$^{ki/wt}$ and mouse angiogenesis genes in the reported dataset (*p*<0.05). *p* value was calculated by Wald test based on Negative Binomial Regression Analysis. (**b**) Information on the qRT–PCR primers used in this study.

Supplementary Table 2

a. Detailed sequence of Mito-Dendra2

	Forward	Reverse
Mito-Dendra2	5’-<br>ATGTCCGTCCTGACGCCGCTGCTGCTGC<br>GGGGCTTGACAGGCTCGGCCCGGCGGC<br>TCCCAGTGCCGCGCGCCAAGATCCATTC<br>GTTGGGGGATCCGAACACCCCGGGAATT<br>AACCTGATCAAGGAGGACATGCGCGTGA<br>AGGTGCACATGGAGGGCAACGTGAACGG<br>CCACGCCTTCGTGATCGAGGGCGAGGG<br>CAAGGGCAAGCCCTACGAGGGCACCCA<br>GACCGCCAACCTGACCGTGAAGGAGGG<br>CGCCCCCCTGCCCTTCAGCTACGACATC<br>CTGACCACCGCCGTGCACTACGGCAACC<br>GGGTGTTCACCAAGTACCCCGAGGACAT<br>CCCCGACTACTTCAAGCAGAGCTTCCCC<br>GAGGGCTACAGCTGGGAGCGCACCATGA<br>CCTTCGAGGACAAGGGCATCTGCACCAT<br>CCGCAGCGACATCAGCCTGGAGGGCGA<br>CTGCTTCTTCCAGAACGTGCGCTTCAAG<br>GGCACCAACTTCCCCCCCAACGGCCCCG<br>TGATGCAGAAGAAGACCCTGAAGTGGGA<br>GCCCAGCACCGAGAAGCTGCACGTGCG<br>CGACGGCCTGCTGGTGGGCAACATCAAC<br>ATGGCCCTGCTGCTGGAGGGCGGCGGC<br>CACTACCTGTGCGACTTCAAGACCACCTA<br>CAAGGCCAAGAAGGTGGTGCAGCTGCCC<br>GACGCCCACTTCGTGGACCACCGCATCG<br>AGATCCTGGGCAACGACAGCGACTACAA<br>CAAGGTGAAGCTGTACGAGCACGCCGTG<br>GCCCGCTACAGCCCCCTGCCCAGCCAG<br>GTGTGG-3’	5’-<br>CCACACCTGGCTGGGCAGGGGGCTGTAG<br>CGGGCCACGGCGTGCTCGTACAGCTTCA<br>CCTTGTTGTAGTCGCTGTCGTTGCCCAGG<br>ATCTCGATGCGGTGGTCCACGAAGTGGG<br>CGTCGGGCAGCTGCACCACCTTCTTGGC<br>CTTGTAGGTGGTCTTGAAGTCGCACAGGT<br>AGTGGCCGCCGCCCTCCAGCAGCAGGG<br>CCATGTTGATGTTGCCCACCAGCAGGCC<br>GTCGCGCACGTGCAGCTTCTCGGTGCTG<br>GGCTCCCACTTCAGGGTCTTCTTCTGCAT<br>CACGGGGCCGTTGGGGGGGAAGTTGGT<br>GCCCTTGAAGCGCACGTTCTGGAAGAAG<br>CAGTCGCCCTCCAGGCTGATGTCGCTGC<br>GGATGGTGCAGATGCCCTTGTCCTCGAA<br>GGTCATGGTGCGCTCCCAGCTGTAGCCC<br>TCGGGGAAGCTCTGCTTGAAGTAGTCGG<br>GGATGTCCTCGGGGTACTTGGTGAACAC<br>CCGGTTGCCGTAGTGCACGGCGGTGGTC<br>AGGATGTCGTAGCTGAAGGGCAGGGGG<br>GCGCCCTCCTTCACGGTCAGGTTGGCGG<br>TCTGGGTGCCCTCGTAGGGCTTGCCCTT<br>GCCCTCGCCCTCGATCACGAAGGCGTGG<br>CCGTTCACGTTGCCCTCCATGTGCACCTT<br>CACGCGCATGTCCTCCTTGATCAGGTTAA<br>TTCCCGGGGTGTTCGGATCCCCCAACGA<br>ATGGATCTTGGCGCGCGGCACTGGGAGC<br>CGCCGGGCCGAGCCTGTCAAGCCCCGC<br>AGCAGCAGCGGCGTCAGGACGGACAT-3’

b. Detailed sequence of *Rhot1* shRNA

	Forward	Reverse
Rhot1 shRNA	5'-<br>CCGGGATATCTCAGAGTCGGAATTTCTC<br>GAGAAATTCCGACTCTGAGATATCTTTTT<br>T-3'	5‘-<br>AAAAAAGATATCTCAGAGTCGGAATTTCT<br>CGAGAAATTCCGACTCTGAGATATCCCGG<br>-3‘

c. Detailed sequence of *Vegfc*, *Slit3*, *Notch3*, *Notch4* siRNA

siRNA	Sense	Antisense
Vegfc	5‘-CCCUAAUUCAUGUGGAGCCAAtt-3‘	5’-UUGGCUCCACAUGAAUUAGGGtt-3‘
Slit3	5’-CGCGAUUUGGAGAUCCUCAtt-3‘	5’-UGAGGAUCUCCAAAUCGCGtt-3‘
Notch3	5‘-GCCAGAACUGUGAAGUCAAtt-3‘	5‘-UUGACUUCACAGUUCUGGCtt-3’
Notch4	5’-GGACUGCUCUGAAGAUAUAtt-3‘	5‘-UAUAUCUUCAGAGCAGUCCtt-3’

Supplementary table 2. (a) Detailed sequence of Mito-Dendra2. (b) Detailed sequence of *Rhot1* shRNA. (c) Detailed sequence of *Vegfc*, *Slit3*, *Notch3*, *Notch4* siRNA.

Supplementary Figure 1

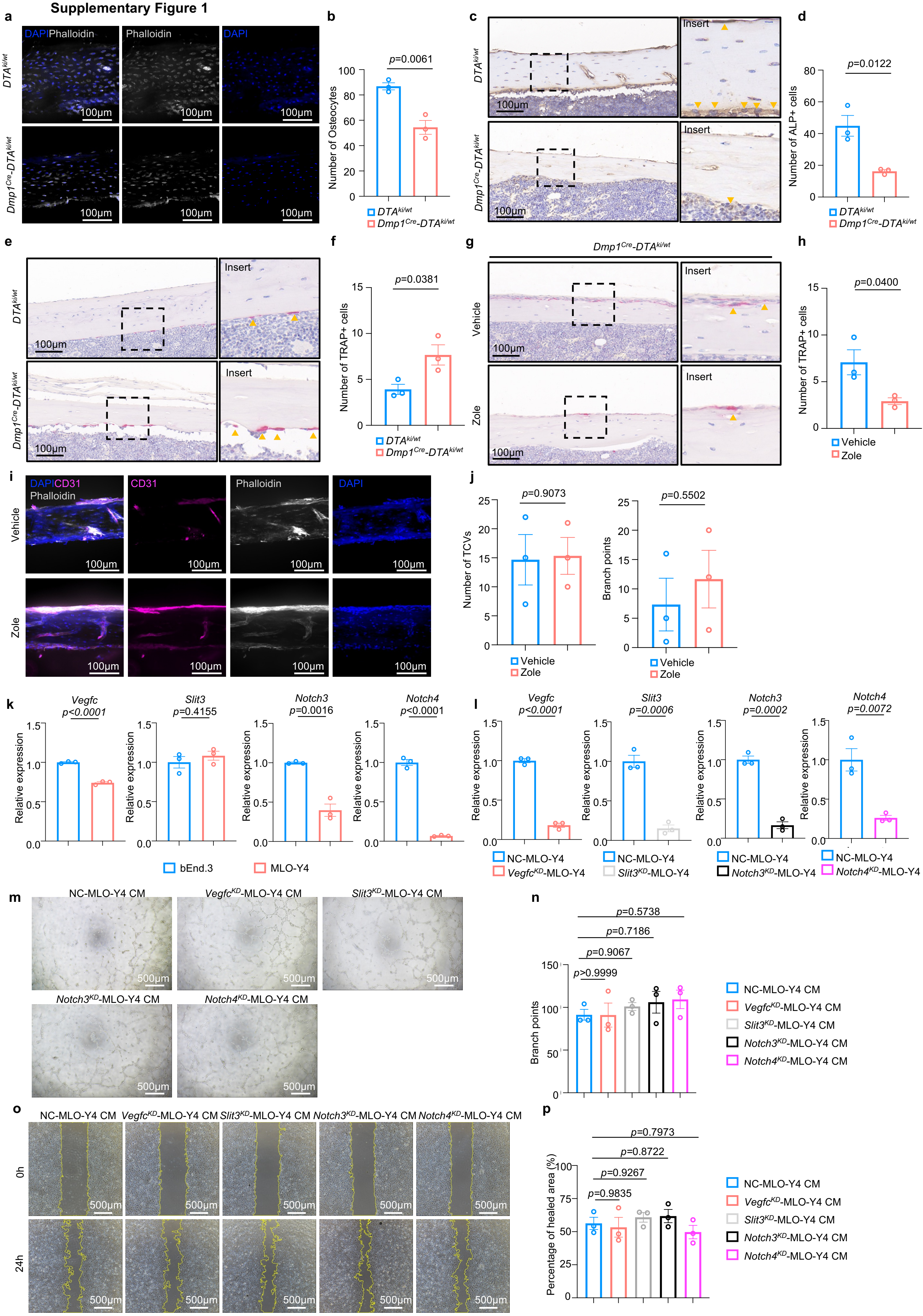

Supplementary Fig. 1. (**a-b**) Representative confocal images of femur cortical bone (**a**) and quantitative result of osteocyte number (**b**) from 4-week-old control *DTA*$^{ki/wt}$ mice and *Dmp1*^{Cre}-*DTA*$^{ki/wt}$ mice. Scale bars, 100μm (n=3 biologically independent samples) (**c-d**) Representative ALP staining images (**c**) of femur cortical bone and quantitative result (**d**) of ALP positive cell number from 4-week-old control *DTA*$^{ki/wt}$ mice and *Dmp1*^{Cre}-*DTA*$^{ki/wt}$ mice. Scale bars, 100μm, yellow arrows represent ALP positive cells (n=3 biologically independent samples). (**e-f**) Representative TRAP staining images (**e**) of femur cortical bone and quantitative result (**f**) of TRAP positive cell number from 4-week-old control *DTA*$^{ki/wt}$ mice and *Dmp1*^{Cre}-*DTA*$^{ki/wt}$ mice. Scale bars, 100μm, yellow arrows represent TRAP positive cells (n=3 biologically independent samples). (**g-h**) Representative TRAP staining images of femur cortical bone (**g**) and quantification of TRAP positive cell number (**h**) from PBS or zoledronic acid (Zole) treated 4-week-old *Dmp1*^{Cre}-*DTA*$^{ki/wt}$mice. Scale bars, 100μm, yellow arrows represent TRAP positive cells (n=3 biologically independent samples). (**i-j**) Representative confocal images of femur cortical bone (**i**) and quantitative result (**j**) of TCVs from PBS or zoledronic acid treated 4-week-old *Dmp1*^{Cre}-*DTA*$^{ki/wt}$ mice. Scale bars, 100μm (n=3 biologically independent samples). (**k**) RT-qPCR analysis of overlapped genes in bEnd.3 cells and MLO-Y4 cells (n=3 biologically independent samples). (**l**) RT-qPCR analysis of targeted gene expression level in *Vegfc*^{KD}-MLO-Y4*,* *Slit3*^{KD}-MLO-Y4*,* *Notch3*^{KD}-MLO-Y4*,* *Notch4*^{KD}-MLO-Y4. "NC" represents "negative control". (n=3 biologically independent samples). (**m-n**) Representative images of tube formation assay (m) and quantitative results (n) of number of branches point of bEnd.3 cells treated with NC-MLO-Y4, *Vegfc*^{KD}-MLO-Y4*,* *Slit3*^{KD}-MLO-Y4*,* *Notch3*^{KD}-MLO-Y4*,* *Notch4*^{KD}-MLO-Y4 conditional media (CM), respectively. Scale bars, 500μm (n=3 biologically independent samples). (**o-p**) Representative images of wound healing assay (**o**) and quantitative result (**p**) of percentage of healing area of bEnd.3 cells treated with NC-MLO-Y4, *Vegfc*^{KD}-MLO-Y4*,* *Slit3*^{KD}-MLO-Y4*,* *Notch3*^{KD}-MLO-Y4*,* *Notch4*^{KD}-MLO-Y4 conditional media (CM), respectively. Scale bars, 500μm (n=3 biologically independent samples). Data were presented as the means ± SEMs. Significance was calculated using unpaired t test with two-tailed P value (**b**, **d**, **f**, **h**, **j**, **k**, **l**), or one-way ANOVA followed by Tukey's post hoc test (**n, p**). Source data are provided as a Source Data file.

Supplementary Figure 2

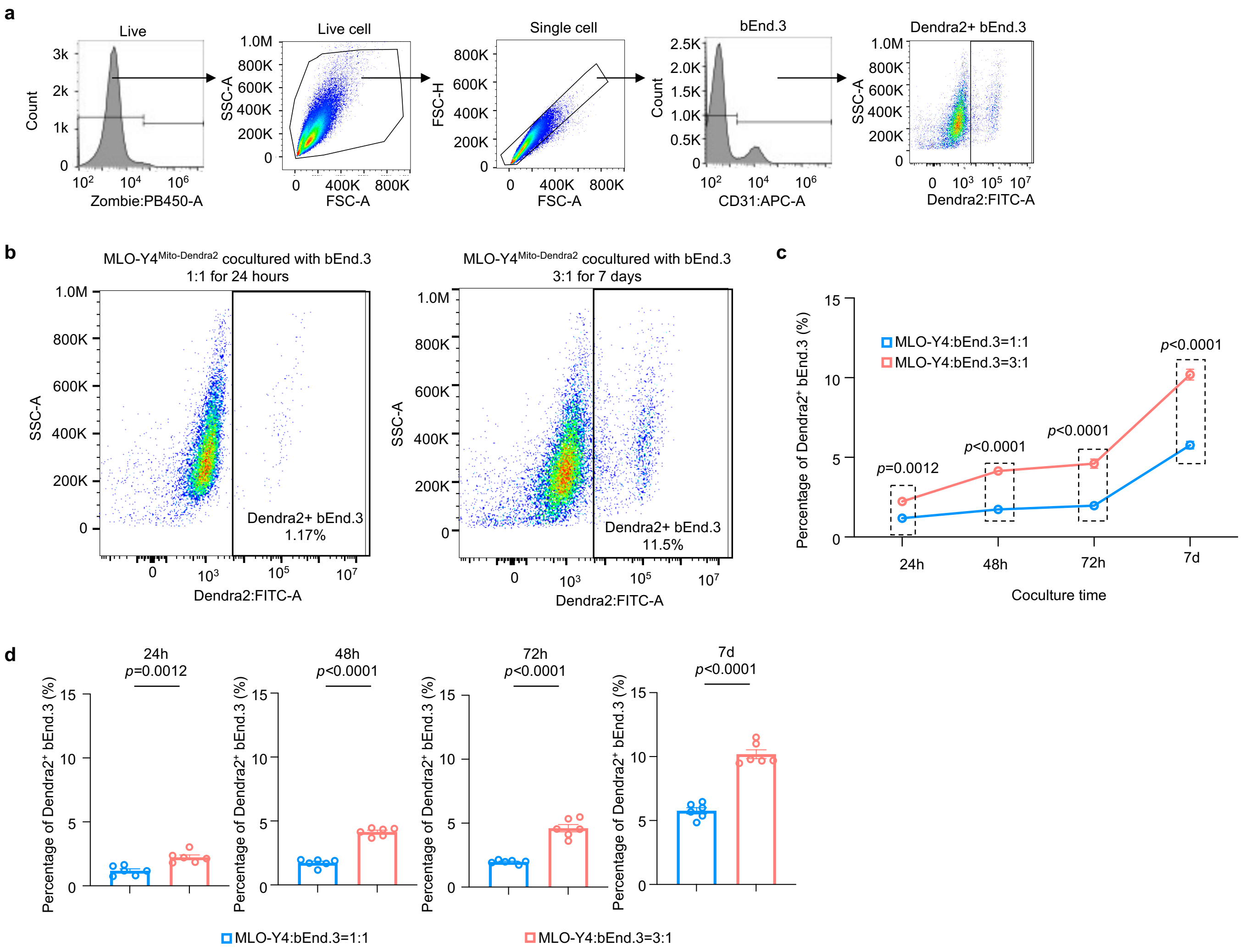

Supplementary Fig. 2. (**a**) Gate strategy to identify Dendra2⁺ bEnd.3 cells cocultured with MLO-Y4$^{Mito\text{-}Dendra2}$ with number ratio at 1:1 and 3:1 (MLO-Y4: bEnd.3) for 24 hours, 48 hours and 72 hours and 7 days. (**b**) Representative dot-plots and quantitative result of the percentage of bEnd.3 cells acquired with Mito-Dendra2 fluorescence in entire bEnd.3 cells population after coculturing with MLO-Y4$^{Mito\text{-}Dendra2}$ for 24 hours with number ratio at 1:1 and for 7 days with number ratio at 3:1 (MLO-Y4: bEnd.3) (**c-d**) Line plot (**c**) and quantitative result (**d**) of bEnd.3 cells acquired with Mito-Dendra2 fluorescence in entire bEnd.3 cells population cocultured with MLO-Y4$^{Mito\text{-}Dendra2}$ with number ratio at 1:1 and 3:1 (MLO-Y4: bEnd.3) for 24 hours, 48 hours and 72 hours and 7 days in (**b**) (n=6 biologically independent samples). Data were presented as the means ± SEMs. Significance was calculated using unpaired t test with two-tailed P value (**c**, **d**). Source data are provided as a Source Data file.

Supplementary Figure 3

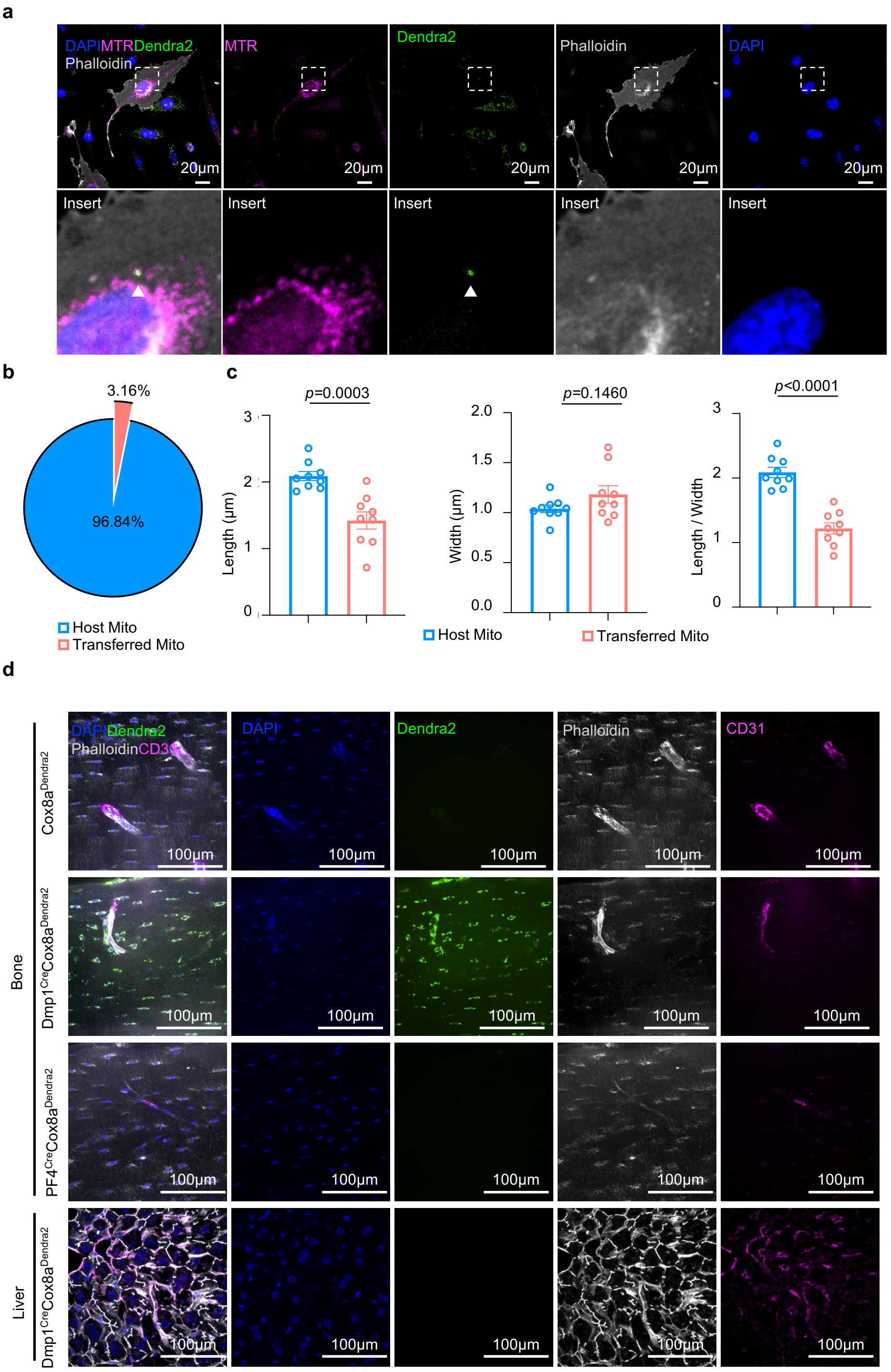

Supplementary Fig. 3. (**a**) Representative confocal images of bEnd.3 cells cocultured with MLO-Y4$^{\text{Mito-Dendra2}}$ with number ratio at 3:1 (MLO-Y4: bEnd.3) for 48 hours. Mitochondria were stained with Mitotracker Red CMXRos (MTR), scale bars, 20μm, white arrow represents MLO-Y4-derived mitochondria (transferred mitochondria). (**b**) Quantitative result of number ratio of bEnd.3 mitochondria (host mito) and MLO-Y4-derived mitochondria (transferred mitochondria) in bEnd.3 cells as shown in (**a**). (n=9 biologically independent samples). (**c**) Quantitative results of morphology of host mitochondria and transferred mitochondria as shown in (**a**) (n=9 biologically independent samples). (**d**) Representative confocal images of femur cortical bone from 4-week-old *Cox8a*^{Dendra2}, *Dmp1*^{Cre}-*Cox8a*^{Dendra2}, *PF4*^{Cre}-*Cox8a*^{Dendra2} and liver from *PF4*^{Cre}-*Cox8a*^{Dendra2} mice. Data were presented as the means ± SEMs. Significance was calculated using unpaired t test with two-tailed P value (**c**). Source data are provided as a Source Data file.

Supplementary Figure 4

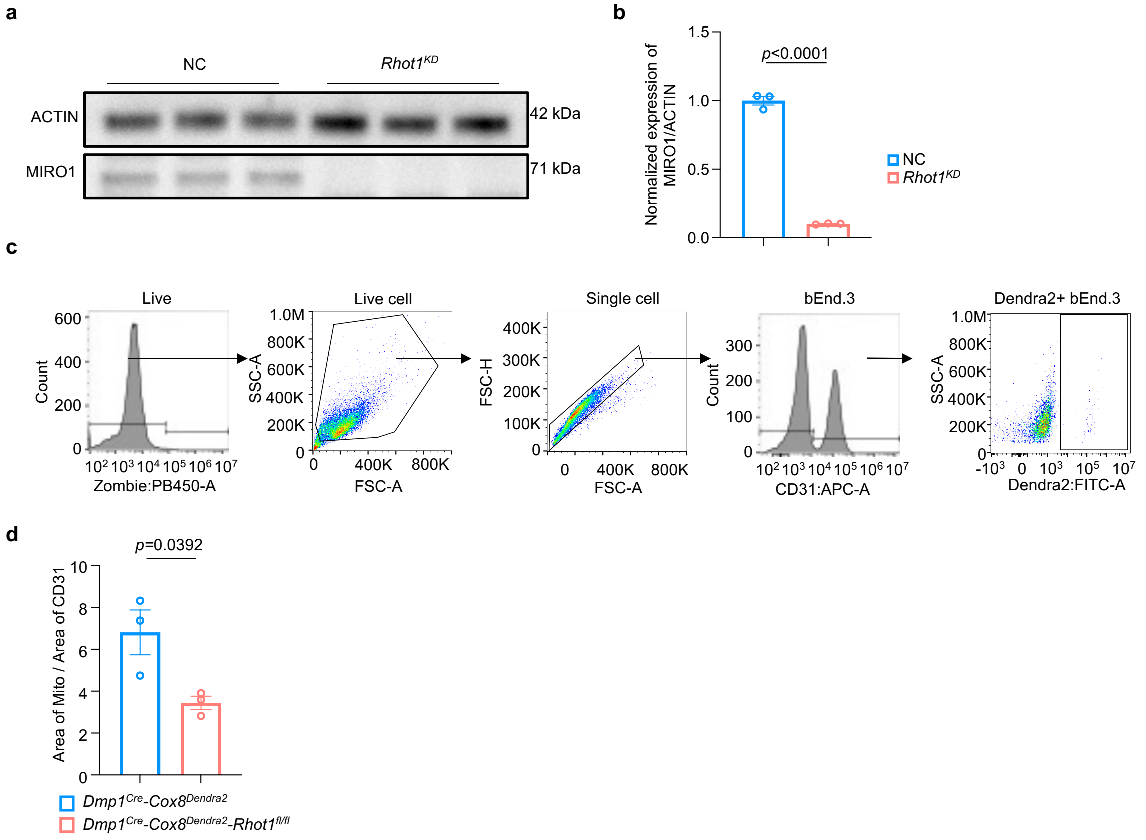

Supplementary Fig. 4. (**a**) Image of Western blot analysis of MIRO1 expression in NC-MLO-Y4 cells and *Rhot1*^{KD}-MLO-Y4 cells. (**b**) Statistical result of *Rhot1* expression as shown in (**a**). (n=3 biologically independent samples). (**c**) Gate strategy to identify bEnd.3 endothelial cells acquired fluorescent mitochondria from NC-MLO-Y4$^{\text{Mito-Dendra2}}$ or *Rhot1*^{KD}-MLO-Y4$^{\text{Mito-Dendra2}}$ after 24 hours of coculture. (**d**) Quantitative result of area of Mito-Dendra2 fluorescence to area of CD31 in femur cortical bone from 6-week-old *Dmp1*^{Cre}-*Cox8a*^{Dendra2} and *Dmp1*^{Cre}-*Cox8a*^{Dendra2}-*Rhot1*$^{fl/fl}$. (n=3 biologically independent samples). Data were presented as the means ± SEMs. Significance was calculated using unpaired t test with two-tailed P value (**b**, **d**). Source data are provided as a Source Data file.

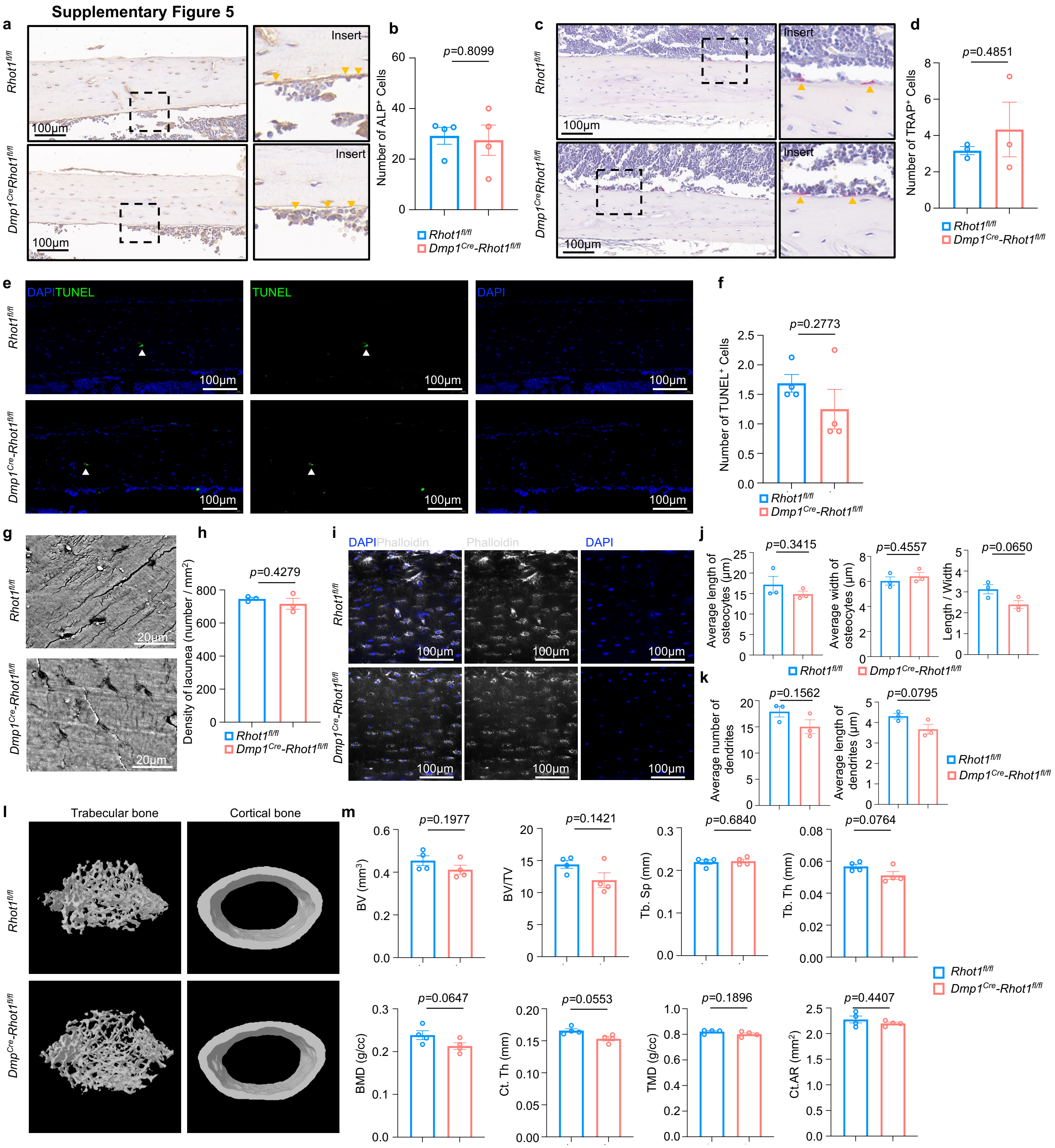

Supplementary Fig. 5. (**a-b**) Representative ALP staining images of femur cortical bone (a) and quantitative result of ALP positive cell number (**b**) from 6-week-old control *Rhot1*$^{fl/fl}$ *and Dmp1*^{Cre}*-Rhot1*$^{fl/fl}$ mice. Scale bars, 100µm, yellow arrows represent ALP positive cells (n=4 biologically independent samples). (**c-d**) Representative TRAP staining images of femur cortical bone (**c**) and quantitative result of TRAP positive cell number (**d**) from 6-week-old control *Rhot1*$^{fl/fl}$ *and Dmp1*^{Cre}*-Rhot1*$^{fl/fl}$ mice. Scale bars, 100µm, yellow arrows represent TRAP positive cells (n=3 biologically independent samples). (**e-f**) Representative TUNEL staining images of femur cortical bone (**e**) and quantitative result of TUNEL positive cell number (**f**) from 6-week-old control *Rhot1*$^{fl/fl}$ *and Dmp1*^{Cre}*-Rhot1*$^{fl/fl}$ mice. Scale bars, 100µm, white arrows represent TUNEL positive cells (n=4 biologically independent samples). (**g-h**) Representative SEM images of femur cortical bone (**g**) and quantitative result of lacunae number (**h**) from 6-week-old control *Rhot1*$^{fl/fl}$ *and Dmp1*^{Cre}*-Rhot1*$^{fl/fl}$ mice. Scale bars, 20µm (n=3 biologically independent samples). (**i-k**) Representative confocal images of femur cortical bone (**i**) and quantitative result of osteocytes morphology (**j**) and dendrites (**k**) from 6-week-old control *Rhot1*$^{fl/fl}$ *and Dmp1*^{Cre}*-Rhot1*$^{fl/fl}$ mice. Scale bars, 100µm (n=3 biologically independent samples). (**l-m**) Representative micro-CT images of femur (**l**) and quantitative result of bone mass (**m**) from 6-week-old control *Rhot1*$^{fl/fl}$ *and Dmp1*^{Cre}*-Rhot1*$^{fl/fl}$ mice (n=4 biologically independent samples). Data were presented as the means ± SEMs. Significance was calculated using unpaired t test with two-tailed P value (**b**, **d**, **f**, **h**, **j**, **m**) or paired t test with two-tailed P value (**m**). Source data are provided as a Source Data file.

Supplementary Figure 6

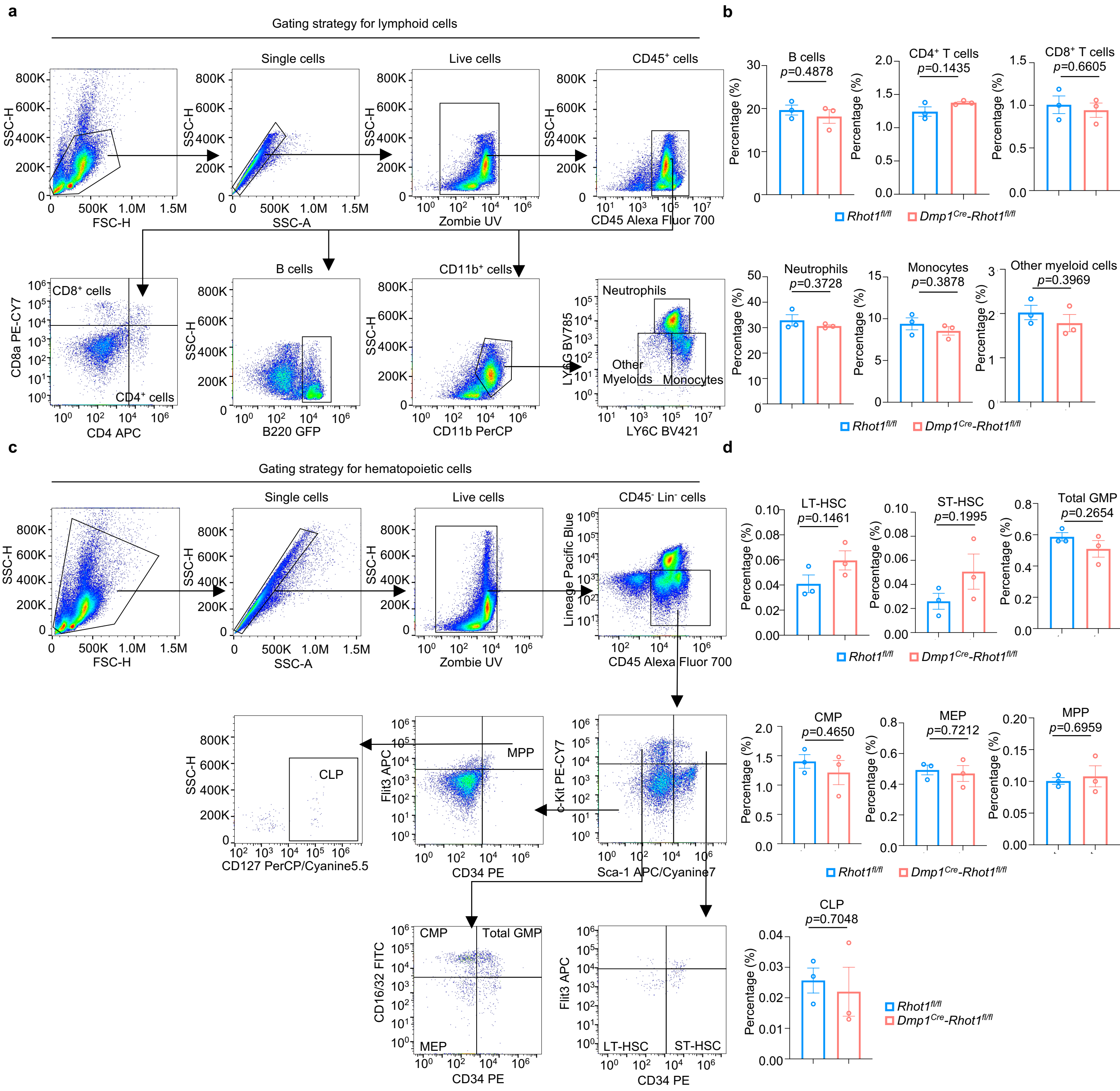

Supplementary Fig. 6. (**a-b**) Gate strategy to identify lymphoid cells (B cells, $CD4^+$ T cells, $CD8^+$ T cells, neutrophils, monocytes, other myeloid cells) (**a**) and quantitative result of cells percentages (**b**) of bone marrow from from 6-week-old control *Rhot1$^{fl/fl}$ and Dmp1^{Cre}-Rhot1$^{fl/fl}$* mice (n=3 biologically independent samples). (**c-d**) Gate strategy to identify hematopoietic cells (LT-HSC, ST-HSC, Total GMP, CMP, MEP, MPP, CLP) (**c**) and quantitative result of cells percentages (**d**) of bone marrow from from 6-week-old control *Rhot1$^{fl/fl}$ and Dmp1^{Cre}-Rhot1$^{fl/fl}$* mice (n=3 biologically independent samples). Data were presented as the means ± SEMs. Significance was calculated using unpaired t test with two-tailed P value (**b**, **d**). Source data are provided as a Source Data file.

Supplementary Figure 7

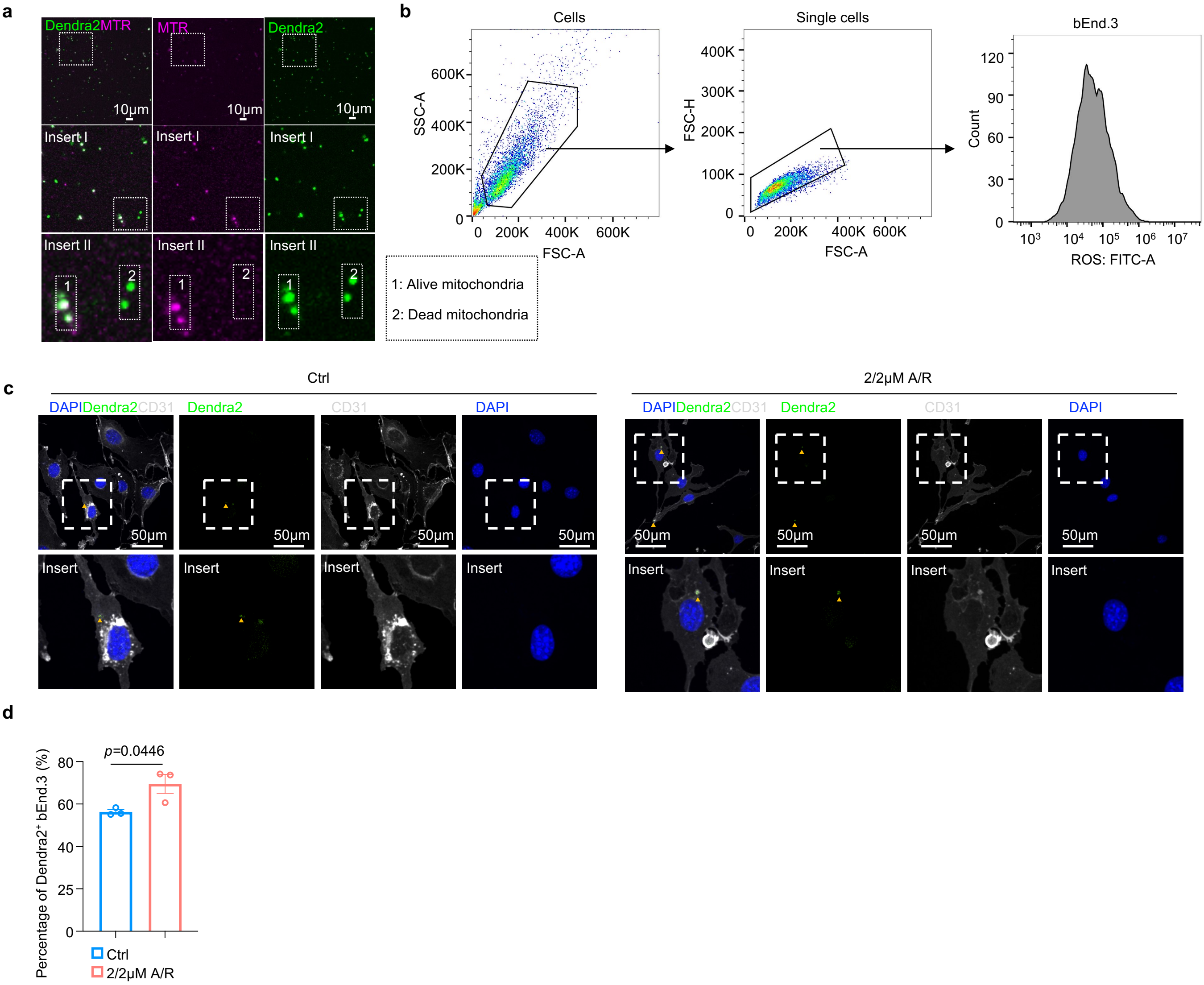

Supplementary Fig. 7. (**a**) Confocal images of mitochondria isolated from MLO-Y4$^{\text{Mito-Dendra2}}$ cells. Box 1: live mitochondria labeled with Dendra2 and MitoTracker Red CMXRos, box 2: dead mitochondria labeled with only Dendra2. Scale bar, 10μm. (**b**) Gate strategy to identify bEnd.3 endothelial cells for measurement of fluorescence intensity of DFCH-DA after transplantation of MLO-Y4$^{\text{Mito-Dendra2}}$ mitochondria. (**c**) Representative confocal images of healthy or 2/2μM antimycin A/rotenone damaged bEnd.3 cells after transplantation of MLO-Y4$^{\text{Mito-Dendra2}}$ mitochondria for 48 hours. scale bars, 50μm, yellow arrows represent transferred mitochondria. (**d**) Quantitative result of bEnd.3 cells acquired with Mito-Dendra2 fluorescence in entire bEnd.3 cells population as shown in (**c**) (n=3 biologically independent samples). Data were presented as the means ± SEMs. Significance was calculated using unpaired t test with two-tailed P value (**d**). Source data are provided as a Source Data file.

Supplementary Figure 8

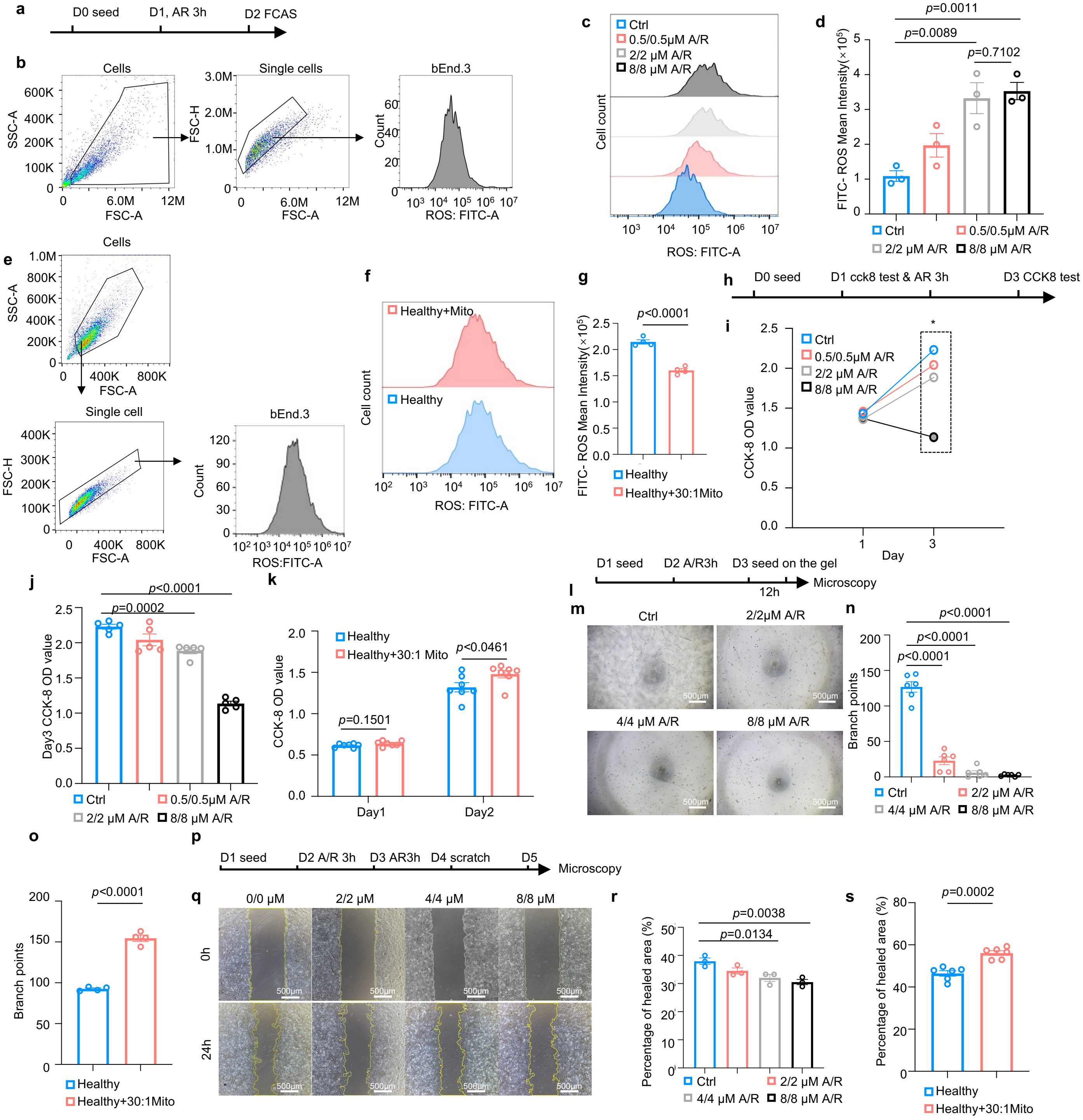

Supplementary Fig. 8. (**a-d**) The influence of antimycin A and rotenone (A/R) on ROS levels in bEnd.3 cells. (**a**) Workflow. (**b**) Gate strategy. (**c-d**) Representative histogram plot of ROS levels (**c**) and statistical result (d) of bEnd.3 cells treated with A/R at concentrations of 0/0μM, 0.5/0.5μM, 2/2μM, and 8/8μM (n=3 biologically independent samples). (**e-g**) The effect of MLO-Y4 cells mitochondria on ROS level on healthy bEnd.3 cells. (**e**) Gate strategy. (**f-g**) Representative histogram plot of ROS levels (**f**) and statistical result (**g**) of bEnd.3 cells treated with MLO-Y4 cells mitochondria (n=4 biologically independent samples). (**h-j**) The influence of A/R on the proliferation rate of bEnd.3 cells. (**h**) Workflow. (**i-j**) The CCK-8 cell proliferation assay on bEnd.3 cells treated with A/R at concentrations of 0/0μM, 0.5/0.5μM, 2/2μM, and 8/8μM throughout 3 days (**i**) and statistical result of CCK-8 OD value on day 3 (**j**) (n=5 biologically independent samples). (**k**) Statistical result of CCK-8 OD value of bEnd.3 cells treated with or without MLO-Y4 cells mitochondria (n=7 biologically independent samples). (**l-n**) The influence of A/R on tube formation of bEnd.3 cells. (**l**) Workflow. (**m-n**) Representative images of tube formation assay (**m**) and quantitative result (**n**) of bEnd.3 cells treated with A/R at concentrations of 0/0μM, 2/2μM, 4/4μM, 8/8μM. Scale bars, 500μm (n=6 biologically independent samples). (**o**) Statistical result of tube formation assay of bEnd.3 cells treated with or without MLO-Y4 cells mitochondria (n=4 biologically independent samples). (**p-r**) The influence of A/R on migration ability of bEnd.3 cells. (**p**) Workflow. (**q-r**) Representative images of wound healing (**q**) and quantitative result (**r**) of bEnd.3 cells treated with A/R at concentrations of 0/0μM, 2/2μM, 4/4μM, 8/8μM. Scale bars, 500μm (n=3 biologically independent samples). (**s**) Quantitative result of wound healing assay of bEnd.3 cells treated with or without MLO-Y4 cells mitochondria (n=6 biologically independent samples). Data were presented as means ± SEMs; Significance was calculated using unpaired t-test with two tailed P value (**d**, **g**, **k**, **o**, **s**) or one way ANOVA followed by Turkey's post hoc test (**j**, **n**, **q**). Source data are provided as a Source Data file.

Supplementary Figure 9

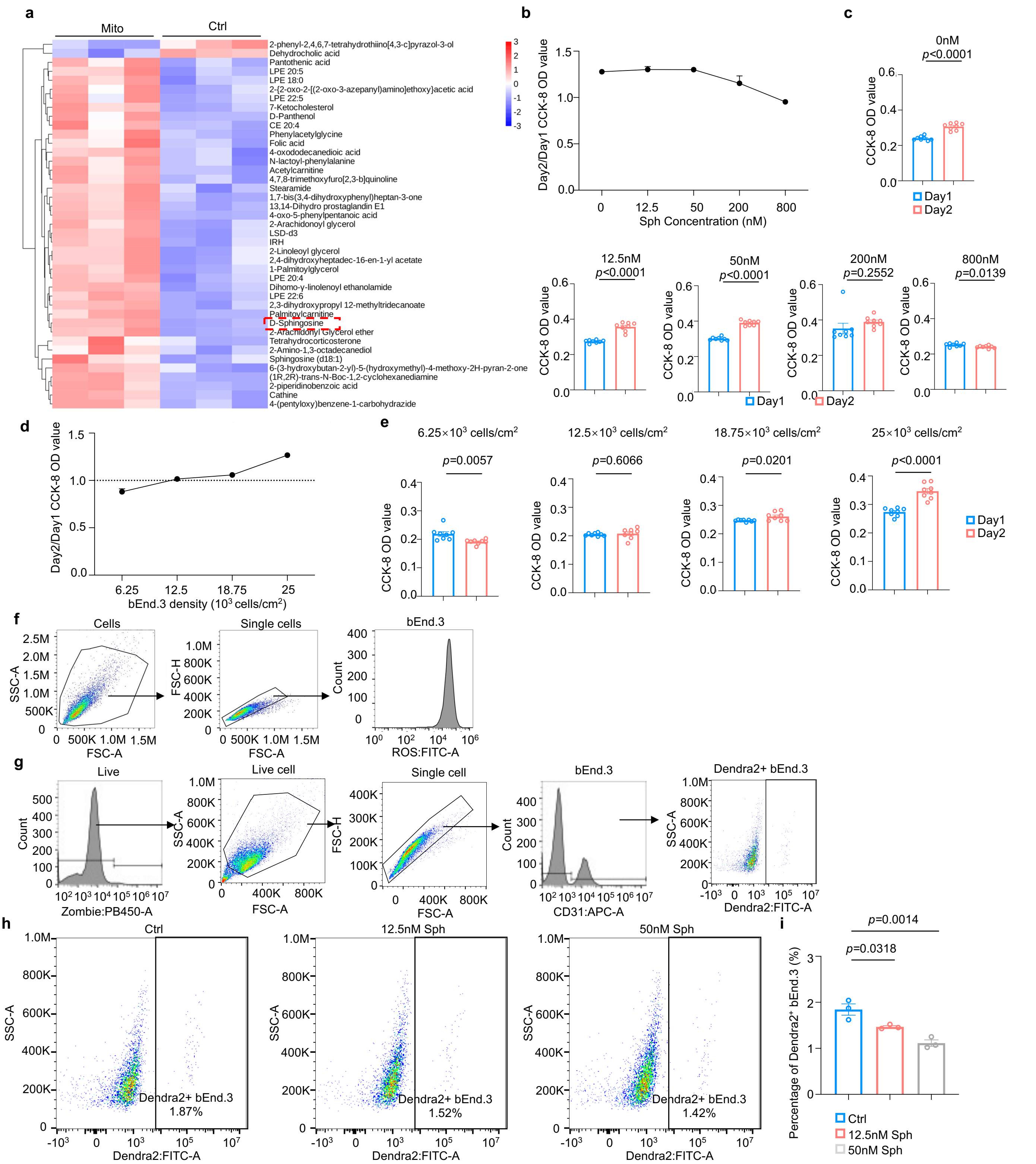

Supplementary Fig. 9. (**a**) Heatmap of untargeted metabolomic analysis show the differentially detected (P<0.05, FC>1.2 or FC<0.833, VIP > 1) metabolites in bEnd.3 cells transplanted with MLO-Y4 mitochondria compared to bEnd.3 cells without mitochondrial transplantation (n=3 biologically independent samples). (**b-c**) Line plot (**b**) and quantitative result (**c**) of CCK-8 assay to investigate the effect of different D-sphingosine concentration on bEnd.3 with seeding density 12500 cell/cm^2 (n=8 biologically independent samples). (**d-e**) Line plot (**d**) and quantitative result (**e**) of CCK-8 assay to investigate the reaction of bEnd.3 to 50nM D-sphingosine with cell seed density at 6250, 12500 and 18750 and 25000 cells/cm^2 (n=8 biologically independent samples). (**f**) Gate strategy to identify bEnd.3 endothelial cells for measurement of fluorescence intensity of DFCH-DA after being treated with different concentrations of D-sphingosine. (**g-i**) The influence of D-sphingosine on bEnd.3 cells acquiring mitochondria from MLO-Y4$^{\text{Mito-Dendra2}}$. (**g**) Gate strategy to identify bEnd.3 endothelial cells acquired fluorescent mitochondria (**h-i**) Representative dot plot (**h**) and quantitively results (**i**) of the percentage of bEnd.3 cells acquired with Mito-Dendra2 fluorescence in entire bEnd.3 cells population that cocultured with MLO-Y4$^{\text{Mito-Dendra2}}$ for 48 hours in present of 12.5nM, 25nM, 50nM D-sphingosine (n=3 biologically independent samples). Data were presented as means ± SEMs; Significance was calculated using unpaired t-test with two tailed P value (**c**, **e**, **i**).

Supplementary Figure 10

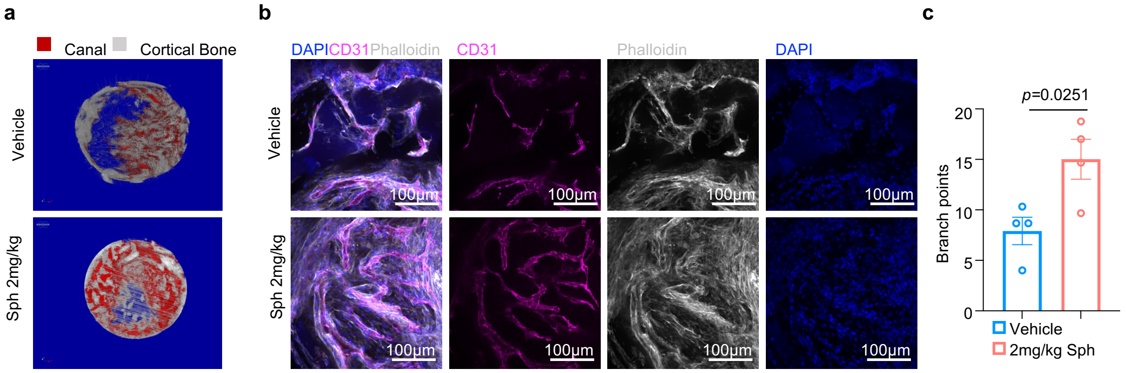

Supplementary Fig. 10. (**a**) Representative images of high-resolution μCT (1μm resolution) on femur callus presenting the naive canal structure in bone defect area from mice that treated with D-sphingosine or saline. (**b-c**) Representative confocal images of callus (**b**) and quantitative result of TCVs (**c**) in bone defect are from mice that treated with D-sphingosine or saline. Scale bars, 100μm (n=4 biologically independent samples). Data were presented as means ± SEMs; Significance was calculated using unpaired t-test with two tailed P value (**c**). Source data are provided as a Source Data file.

Supplementary Figure 11 Uncropped Western blots.

Supplementary Fig. 4a

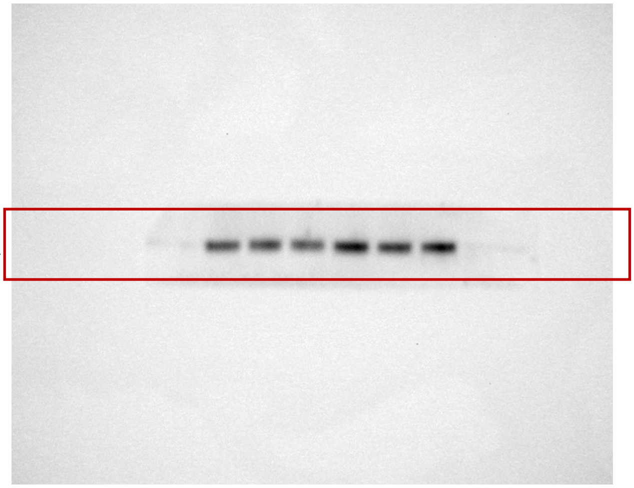

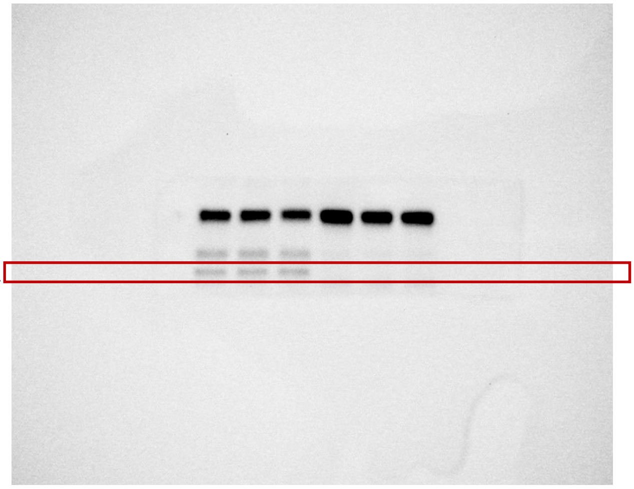

Supplement: Supplementary file 1 — Supplementary Information [file 41467_2024_46095_MOESM1_ESM.pdf]
