# Supplementary material for: Osteocyte mitochondria regulate angiogenesis of transcortical vessels
Source: Nat Commun. 2024 Mar 21;15:2529. doi: 10.1038/s41467-024-46095-0 (PMC10957947; doi:10.1038/s41467-024-46095-0)
Supplement: Supplementary file 3 — Reporting Summary [file 41467_2024_46095_MOESM3_ESM.pdf]

nature portfolio

Corresponding author(s): Junjie Gao

Last updated by author(s): Nov 29, 2023

Reporting Summary

Nature Portfolio wishes to improve the reproducibility of the work that we publish. This form provides structure for consistency and transparency in reporting. For further information on Nature Portfolio policies, see our Editorial Policies and the Editorial Policy Checklist.

Statistics

For all statistical analyses, confirm that the following items are present in the figure legend, table legend, main text, or Methods section.

n/a	Confirmed	
☐	☒	The exact sample size (*n*) for each experimental group/condition, given as a discrete number and unit of measurement
☐	☒	A statement on whether measurements were taken from distinct samples or whether the same sample was measured repeatedly
☐	☒	The statistical test(s) used AND whether they are one- or two-sided<br>*Only common tests should be described solely by name; describe more complex techniques in the Methods section.*
☒	☐	A description of all covariates tested
☐	☒	A description of any assumptions or corrections, such as tests of normality and adjustment for multiple comparisons
☐	☒	A full description of the statistical parameters including central tendency (e.g. means) or other basic estimates (e.g. regression coefficient) AND variation (e.g. standard deviation) or associated estimates of uncertainty (e.g. confidence intervals)
☐	☒	For null hypothesis testing, the test statistic (e.g. *F*, *t*, *r*) with confidence intervals, effect sizes, degrees of freedom and *P* value noted<br>*Give P values as exact values whenever suitable.*
☒	☐	For Bayesian analysis, information on the choice of priors and Markov chain Monte Carlo settings
☒	☐	For hierarchical and complex designs, identification of the appropriate level for tests and full reporting of outcomes
☒	☐	Estimates of effect sizes (e.g. Cohen's *d*, Pearson's *r*), indicating how they were calculated

Our web collection on statistics for biologists contains articles on many of the points above.

Software and code

Policy information about availability of computer code

Data collection

The software used for data collection are listed as follows:
Leica Application Suite X 3.5.5.19976 or Zeiss ZEN pro or Olympus cellSens Dimension 3.2 software were used for capturing images at wide field or confocal micrsocopes.
Nikon Nis Elements 5.11 was used for capturing images at bright filed.
MPM 6.0 was used for collecting absorb value of CCK8.
Skanlt Software for Microplate Readers 6.1.0.51 was used for collecting absorb value of ELISA.
QuantStudio Real-Time PCR 1.3 Software was used for collecting quantitative real-time PCR data.
Image Lab Touch Software 2.3.0.07 was used for visualizing the Western blot.
CytExpert 2.3.1.22 was used for collecting FACS data.
Thermo Fisher Scientific Vanquish UHPLC system coupled with an Orbitrap Q ExactiveTM HF mass spectrometer in LCSW were used to perform UHPLC–MS/MS analyses.
Skycan 1.6.10.4 was used for micro-CT scanning.

Data analysis	The software used for data analysis are listed as follows:<br>For statistical analysis, GraphPad Prism version 9.0, statistical software R (R version R3.4.3), Python (Python 2.7.6 version) and were used.<br>For image analysis, Image J Fiji 2.0.0-rc-69/1.52n and Adobe photoshop 22.4.2 20210602.r.242 a4f6042 x64 were used.<br>For FACS analysis, , FlowJo 10.4 was used.<br>For micro-CT reconstruction, CTAn 1.16.1.0+ and CTvox 3.3.1 were used.<br>For analyzing metabolites, Thermo Fisher Scientific Compound Discoverer 3.1 was used.<br>For Principal component analysis and partial least squares discriminant analysis, metaX was used.<br>No custom codes were used during this study.

For manuscripts utilizing custom algorithms or software that are central to the research but not yet described in published literature, software must be made available to editors and reviewers. We strongly encourage code deposition in a community repository (e.g. GitHub). See the Nature Portfolio guidelines for submitting code & software for further information.

Data

Policy information about availability of data

All manuscripts must include a data availability statement. This statement should provide the following information, where applicable:

- Accession codes, unique identifiers, or web links for publicly available datasets
- A description of any restrictions on data availability
- For clinical datasets or third party data, please ensure that the statement adheres to our policy

Bulk RNA-seq datasets have been deposited in the Gene Expression Omnibus (GEO) database under accession codes GSE20235614. Metabolomics data have been deposited in the Metabolights database, with accession numbers MTBLS7332 (Release date: 2024-02-28). Source data are provided with this paper.

Research involving human participants, their data, or biological material

Policy information about studies with human participants or human data. See also policy information about sex, gender (identity/presentation), and sexual orientation and race, ethnicity and racism.

Reporting on sex and gender	No human studies were performed.
Reporting on race, ethnicity, or other socially relevant groupings	No human studies were performed.
Population characteristics	No human studies were performed.
Recruitment	No human studies were performed.
Ethics oversight	No human studies were performed.

Note that full information on the approval of the study protocol must also be provided in the manuscript.

Field-specific reporting

Please select the one below that is the best fit for your research. If you are not sure, read the appropriate sections before making your selection.

☒ Life sciences ☐ Behavioural & social sciences ☐ Ecological, evolutionary & environmental sciences

For a reference copy of the document with all sections, see nature.com/documents/nr-reporting-summary-flat.pdf

Life sciences study design

All studies must disclose on these points even when the disclosure is negative.

Sample size	No statistical methods were used to predetermine sample size but rather, all experiments were performed with at least biological triplicate to ensure reproducibility. For in vivo study, we have 3-9 mice in each group (Further detailed can be found in figure legend). For in vitro study, we have at least 3-9 biological replicates. No sample size calculation were performed because sample size was determined based on the standard in the field for the type of experiment being performed.
Data exclusions	No data was excluded from the analysis.
Replication	All in vitro study, we have at least 3 biological replicates. For in vivo study, we have 3-9 mice in each group. All attempts at replication were successful.
Randomization	The samples including used in this study were randomly allocated into control or experimental groups
Blinding	To ensure the blindness of data collection and analysis, samples for metabolic profiling were numbered independently. Metabolomics were performed by technicians at Hangzhou LC-Bio Technology company who are blinded to the sample information. For other experiments the

investigators were not blinded since analyses relied on unbiased measurements of quantitative parameters. However, standardized procedures for data collection and analysis were used to prevent bias.

Reporting for specific materials, systems and methods

We require information from authors about some types of materials, experimental systems and methods used in many studies. Here, indicate whether each material, system or method listed is relevant to your study. If you are not sure if a list item applies to your research, read the appropriate section before selecting a response.

Materials & experimental systems

n/a	Involved in the study
☐	☒ Antibodies
☐	☒ Eukaryotic cell lines
☒	☐ Palaeontology and archaeology
☐	☒ Animals and other organisms
☒	☐ Clinical data
☒	☐ Dual use research of concern
☒	☐ Plants

Methods

n/a	Involved in the study
☒	☐ ChIP-seq
☐	☒ Flow cytometry
☒	☐ MRI-based neuroimaging

Antibodies

Antibodies used

Only commercial antibodies have been used.All the antibodies are from commercial sources and have been validated by the vendors and their validation data are available on the manufacturer's website.
Anti-CD31 antibody (R&D systems, Cat. AF3628), 1:200 on bone samples, 1:100 on cell samples.
APC anti-mouse CD31 Antibody (Biolegend, Cat. 102510), 1:200
Anti-SPHK1 antibody (Affinity, Cat. DF6005), 1:100 for IF, 1:500 for WB
Anti-Miro1 antibody (Abcam, Cat. ab211363), 1:1000
β-Actin antibody, (Cell Signaling Technology, Cat. 4970s), 1:1000
Anti-Tom20 antibody, (Cell Signaling Technology, Cat. 42406s), 1:1000
Anti-VDAC antibody, (Cell Signaling Technology, Cat. 4661s), 1:1000
Lamin A/C Antibody, (Cell Signaling Technology, Cat. 2032s), 1:1000
Rabbit polyclonal antibody to Alkaline Phosphatase (Affinity, Cat.DF6225), 1:100
Donkey anti-goat IgG (H+L) cross-adsorbed secondary antibody, Alexa Fluor 647, (Thermo Fisher Scientific, Cat. A21447), 1:200
Donkey anti-goat IgG (H+L) cross-adsorbed secondary antibody, Alexa Fluor 568, (Thermo Fisher Scientific, Cat. A11057), 1:200
Donkey anti-rabbit IgG (H+L) highly cross-adsorbed secondary antibody, Alexa Fluor 647 (Thermo Fisher Scientific, Cat. A31573), 1:200

Validation

All antibodies used in this study were obtained from commercial sources and validated according to manufacturers's instruction.
Anti-CD31 antibody (R&D systems, Cat. AF3628): 231 citations reported on manufacturer's website: https://www.rndsystems.com/products/human-mouse-rat-cd31-pecam-1-antibody_af3628#product-citations
APC anti-mouse CD31 Antibody (Biolegend, Cat. 102510): 56 citations reported on manufacturer's website: https://www.biolegend.com/de-de/products/apc-anti-mouse-cd31-antibody-375?GroupID=BLG10531
Anti-SPHK1 antibody (Affinity, Cat. DF6005): 3 citations reported on manufacturer's website: https://www.affbiotech.com/goods-4807-DF6005-SPHK1_Antibody.html
Anti-Miro1 antibody (Abcam, Cat. ab211363): Myo19 tethers mitochondria to endoplasmic reticulum-associated actin to promote mitochondrial fission. Cell Sci. 2023;136(5):jcs260612. doi:10.1242/jcs.260612
β-Actin antibody, (Cell Signaling Technology, Cat. 4970s): 5795 citations reported on manufacturer's website: https://www.cellsignal.com/products/primary-antibodies/b-actin-13e5-rabbit-mab/4970
Anti-Tom20 antibody, (Cell Signaling Technology, Cat. 42406s): 285 citations reported on manufacturer's website: https://www.cellsignal.com/products/primary-antibodies/tom20-d8t4n-rabbit-mab/42406
Anti-VDAC antibody, (Cell Signaling Technology, Cat. 4661s): 325 citations reported on manufacturer's website: https://www.cellsignal.com/products/primary-antibodies/vdac-d73d12-rabbit-mab/4661
Lamin A/C Antibody, (Cell Signaling Technology, Cat. 2032s): 324 citations reported on manufacturer's website: https://www.cellsignal.com/products/primary-antibodies/lamin-a-c-antibody/2032
Rabbit polyclonal antibody to Alkaline Phosphatase (Affinity, Cat.DF6225): 17 citations reported on manufacturer's website: https://www.affbiotech.cn/goods-5027-DF6225-Alkaline_Phosphatase_Antibody.html#acc_citation
Donkey anti-goat IgG (H+L) cross-adsorbed secondary antibody, Alexa Fluor 647, (Thermo Fisher Scientific, Cat. A21447): 1152 citations reported on manufacturer's website: https://www.thermofisher.com/antibody/product/Donkey-anti-Goat-IgG-H-L-Cross-Adsorbed-Secondary-Antibody-Polyclonal/A-21447
Donkey anti-goat IgG (H+L) cross-adsorbed secondary antibody, Alexa Fluor 568, (Thermo Fisher Scientific, Cat. A11057): 860 citations reported on manufacturer's website: https://www.thermofisher.com/antibody/product/Donkey-anti-Goat-IgG-H-L-Cross-Adsorbed-Secondary-Antibody-Polyclonal/A-11057
Donkey anti-rabbit IgG (H+L) highly cross-adsorbed secondary antibody, Alexa Fluor 647 (Thermo Fisher Scientific, Cat. A31573): 2214 citations reported on manufacturer's website: https://www.thermofisher.com/antibody/product/Donkey-anti-Rabbit-IgG-H-L-Highly-Cross-Adsorbed-Secondary-Antibody-Polyclonal/A-31573

Eukaryotic cell lines

Policy information about cell lines and Sex and Gender in Research

Cell line source(s)	bEnd.3 cell line was obtained from ATCC, Catalog number CRL-2299. MLO-Y4 cell line was a gift from Dr Lynda F Bonewald.
Authentication	bEnd.3 Cell lines were authenticated by short tandem repeat profiling prior to use. MLO-Y4 cell line were authenticated using morphology and short tandem repeat profiling prior to use.
Mycoplasma contamination	All cell lines were tested for mycoplasma at several times during this research and only used if the results were negative
Commonly misidentified lines (See ICLAC register)	The cell line used in this study is not present in the registry of commonly misidentified lines

Animals and other research organisms

Policy information about studies involving animals; ARRIVE guidelines recommended for reporting animal research, and Sex and Gender in Research

Laboratory animals	4-13 weeks mice were used for experiments. C57BL/6J (WT) (stock number: N000013) and mGmT [B6; JGpt-H11em1Cln(CAG-LoxP-ZsGreen-Stop-LoxP-tdTomato)/Gpt, stock number: T006163] mouse strains were purchased from GemPharmatech Co. Ltd. Ltd. (Jiangsu, China). The Dmp1cre transgenic mice were provided by J. Q. (Jerry) Feng at Texas A&M College of Dentistry, USA44. Cox8Dendra2 [B6; 129SGt(ROSA)26Sortm1(CAG-COX8/Dendra2)Dcc/J, stock number: 0183885] and Rhotfl/fl [B6(Cg)-Rhot1tm2.1Jmsu/J, stock number: 031126] mouse strains were purchased from the Jackson Laboratory. An osteocyte ablation mouse model was established by crossing Dmp1Cre mice with Rosa26em1Cin(SA-IRES-Loxp-ZsGreen-stop-Loxp-DTA) homozygotes to obtain Dmp1cre-Rosa26em1Cin(SA-IRES-Loxp-ZsGreen-stop-Loxp-DTA) heterozygotes (DTAki/wt). The mice were group-housed with at most 5 per cage in a stable environment (23–25°C ambient temperature and 50% humidity). They were maintained under a 12-h light–dark cycle (lights on from 7:00 to 19:00) with free access to water and food ad libitum. Mice were fed with normal diet with 11.1% energy from fat, 22.9% from protein and 66% from carbohydrates. This study was performed on male mice to exclude the potential influence of estrogen on bone homeostasis.
Wild animals	No wild animals were used in this study.
Reporting on sex	This study was performed on male mice to exclude the potential influence of estrogen on bone homeostasis.
Field-collected samples	No field-collected samples were used in this study.
Ethics oversight	Mice were group-housed in a specific pathogen-free (SPF) facility before being selected for experiments with standard conditions. All procedures regarding animal maintenance and experiments were carried out in accordance with the Institutional Animal Care and Use Committee (IACUC) and approved by the Animal Care and Use Committee of Shanghai Jiao tong University Affiliated Sixth People's Hospital (No.2021-0788).

Note that full information on the approval of the study protocol must also be provided in the manuscript.

Flow Cytometry

Plots

Confirm that:

☒ The axis labels state the marker and fluorochrome used (e.g. CD4-FITC).

☒ The axis scales are clearly visible. Include numbers along axes only for bottom left plot of group (a 'group' is an analysis of identical markers).

☐ All plots are contour plots with outliers or pseudocolor plots.

☒ A numerical value for number of cells or percentage (with statistics) is provided.

Methodology

Sample preparation	For bEnd.3 co-cultured with MLO-Y4 mito-dendra2: Cells were prepared for experiment on 12-well plate and trypsinized for detachment. The cells were collected, washed and resuspended in 2%FBS/PBS. Cells were stained with APC-conjugated CD31 (1:200). Cells were washed twice in 2%FBS/PBS, then resuspended in 200µl 2%FBS/PBS. Samples were analyzed on CytoFLEX Flow Cytometer and data analyzed in FLowjo 10.4<br><br>For ROS measurement, bEnd.3 cells were prepared in 12-well plates and were treated with DMEM containing 10 µM DCFH-DA for 20 minutes at 37°C in a 5% CO2 humidified incubator. Next, the cells were washed with PBS 1 time and centrifuged at 300 × g for 5 minutes. Cell pellets were then resuspended in 200 µl PBS containing 2% FBS and immediately analyzed with a CytoFLEX Flow Cytometer. The data analyzed in FLowjo 10.4
Instrument	Cells were analyzed by Beckman CytoFLEX Flow Cytometer

Software	CytExpert 2.3.1.22 was used for collecting FACS data. Data was then analyzed using FlowJo software v10.4.
Cell population abundance	At least 10 thousands cells were analyzed. We followed standards in the field.
Gating strategy	The gate on FSC-A vs SSC-A was set to include all live cell populations but exclude debris. Doublets and cell aggregates were excluded by gating FSC-A/FSC-H.<br>APC anti-mouse CD31 Antibody (Biolegend, Cat. 102510) was used to identify bEnd.3 from MLO-Y4 (Mito-Dendra2) cells. See Supplementary Information 1 for more details.

☒ Tick this box to confirm that a figure exemplifying the gating strategy is provided in the Supplementary Information.
